# *TP53* Polymorphisms and Colorectal Cancer Risk in Patients with Lynch Syndrome in Taiwan: A Retrospective Cohort Study

**DOI:** 10.1371/journal.pone.0167354

**Published:** 2016-12-01

**Authors:** Abram Bunya Kamiza, Ling-Ling Hsieh, Reiping Tang, Huei-Tzu Chien, Chih-Hsiung Lai, Li-Ling Chiu, Tsai-Ping Lo, Kuan-Yi Hung, Jeng-Fu You, Wen-Chang Wang, Chao A. Hsiung, Chih-Ching Yeh

**Affiliations:** 1 School of Public Health, College of Public Health and Nutrition, Taipei Medical University, Taipei, Taiwan; 2 Department of Public Health, College of Medicine, Chang Gung University, Tao-Yuan, Taiwan; 3 Colorectal Section, Department of Surgery, Chang Gung Memorial Hospital, Tao-Yuan, Taiwan; 4 School of Medicine, Chang Gung University, Tao-Yuan, Taiwan; 5 Department of Nutrition and Health Sciences, Chang Gung University of Science and Technology, Tao-Yuan, Taiwan; 6 Institute of Population Health Sciences, National Health Research Institutes, Miaoli, Taiwan; 7 Ph.D. Program for Translational Medicine, College of Medical Science and Technology, Taipei Medical University, Taipei, Taiwan; 8 Department of Public Health, China Medical University, Taichung, Taiwan; National Cancer Centre Singapore, SINGAPORE

## Abstract

**Background and Aim:**

*TP53* encodes p53, which has a crucial role in modulating genes that regulate defense against cancer development. This study investigated whether *TP53* polymorphisms are associated with colorectal cancer (CRC) in patients with Lynch syndrome and whether *TP53* interacts with lifestyle factors to modify CRC risk.

**Methods:**

We identified 260 *MLH1* and *MSH2* germline mutation carriers from the Taiwan Hereditary Nonpolyposis Colorectal Cancer Consortium. A weighted Cox proportional hazard model was used to calculate hazard ratios (HRs) and 95% confidence intervals (CIs) to determine the association of *TP53* polymorphisms with CRC development.

**Results:**

The carriers of the variant C allele of rs1042522 were associated with a decreased CRC risk (GC genotype: HR = 0.35, 95% CI = 0.14–0.86; CC genotype: HR = 0.28, 95% CI = 0.13–0.57). In addition, the dominant model of rs1042522 was associated with a decreased CRC risk (HR = 0.32, 95% CI = 0.15–0.67). The CRC risk was decreased in carriers with the CT and TT genotypes of rs12947788 (HR = 0.20, 95% CI = 0.08–0.46 and HR = 0.25, 95% CI = 0.09–0.65, respectively). Moreover, the dominant model of rs12947788 was significantly associated with a decreased CRC risk (HR = 0.21, 95% CI = 0.09–0.46). A haplotype analysis indicated that compared with the most common GC haplotype, the CT haplotype was associated with a decreased CRC risk (HR = 0.26, 95% CI = 0.11–0.59). However, no significant interaction was observed between *TP53* polymorphisms and lifestyle factors.

**Conclusion:**

The study results revealed that the rs1042522 genotype with the C allele and the rs12947788 genotype with the T allele in *TP53* were associated with a decreased CRC risk in patients with Lynch syndrome in Taiwan.

## Introduction

Lynch syndrome is a hereditary disorder caused by mutations in mismatch repair (MMR) genes, particularly in *MLH1* and *MSH2* and less commonly in *MSH6* and *PMS2* [1, 2]. Patients with Lynch syndrome have an increased risk of colorectal cancer (CRC) and other cancers [3, 4]. In the Chinese population, the cumulative lifetime risk of CRC in patients with *MLH1* and *MSH2* germline mutations at the age of 70 years is 81.7% and 93.1%, respectively [5]. However, not all people with MMR gene germline mutations develop CRC; this is partially attributable to tumor suppressor genes [6, 7].

*TP53*, one of the most crucial tumor suppressor genes, has a critical role of modulating genes that regulate defense against cancer development through inhibiting the cell cycle, angiogenesis, and cellular senescence and causing apoptosis [8]. *TP53* is located at the point of convergence of several distinct stress response pathways. When DNA in a cell is damaged, p53 plays a critical role in determining whether the damaged DNA will undergo the repair or apoptosis mechanism [9, 10]. This process prevents further division of cells with mutated or damaged DNA, which facilitates the prevention of tumorigenesis [7].

The association of *TP53* polymorphisms with CRC risk in patients with Lynch syndrome has rarely been investigated. Moreover, the results reported thus far have been inconsistent, and several studies have mainly included patients with sporadic CRC [11, 12, 13, 14, 15, 16]. Talseth et al. reported that *TP53* polymorphisms were not associated with CRC development in the Australian and Polish Lynch syndrome populations [17]. Furthermore, Sotamaa et al. reported that *TP53* polymorphisms may not be associated with CRC development in patients with Lynch syndrome [18]; this finding is consistent with that of Talseth et al. However, a cohort study conducted in the United States reported that patients with Lynch syndrome carrying the heterozygous genotype rs1042522 developed CRC 13 years earlier in life than those carrying the homozygous wild-type genotype did [19].

A knowledge gap exists in sporadic CRC studies because of inconsistency in the results of Lynch syndrome studies. Therefore, this study investigated whether *TP53* polymorphisms are associated with CRC risk in patients with Lynch syndrome and whether *TP53* polymorphisms interact with lifestyle factors and modify CRC risk in patients with Lynch syndrome.

## Materials and Methods

### Participant recruitment

Patients were enrolled in the Amsterdam criteria family registry of the Hereditary Nonpolyposis Colorectal Cancer Consortium of the Taiwan National Health Research Institute by using the Amsterdam II Criteria, as described in detail previously [20, 21]. From May 2002 onwards, the clinical data of all index patients with Lynch syndrome were collected. All index patients from seven participating hospitals and medical centers throughout Taiwan were screened for MMR gene germline mutations in *MLH1* and *MSH2*. In addition, the index patients were requested to contact their family members to seek permission for their enrollment in the registry.

The study was conducted in accordance with the 1975 Declaration of Helsinki, and the research protocol was approved by the Institutional Review Board of Taipei Medical University and the Taiwan National Health Research Institutes. Written informed consent was obtained from each study participant. In total, 1014 family members from 135 Amsterdam II Criteria families were registered, and their genetic analyses were completed by February 2012, as described in detail previously [21]. We identified 303 germline mutation carriers from the Taiwan Hereditary Nonpolyposis Colorectal Cancer Consortium. Two patients were excluded because they harbored mutations in both *MLH1* and *MSH2*. In addition, 41 patients were excluded because they did not have information on *TP53* polymorphisms. We finally recruited 260 confirmed MMR gene germline mutation carriers from 62 families.

### Data collection

All probands and their relatives were interviewed by professional nurses in the colorectal surgery department of participating hospitals. The nurses were trained to conduct interviews and had no prior knowledge of study hypotheses regarding genetic factors, lifestyle factors, dietary intake factors, and CRC. All interviews were uniformly administered in wards by using a structured questionnaire covering questions on sociodemographic variables, lifestyle factors (cigarette smoking and alcohol, tea, and coffee consumption), and medical history.

The status of cigarette smoking and alcohol, tea, and coffee consumption was categorized as never, former, or current as described in our previous study [21]. We observed that few MMR gene germline mutation carriers were former cigarette smokers or alcohol, tea, or coffee consumers. Therefore, we combined the former and current users to form the “ever user” category. We biennially followed up all the participants from May 2002 to February 2012 to obtain updates about their morbidity status. The cancer diagnosis and age at diagnosis were confirmed, wherever possible, on the basis of pathology reports, medical records, cancer registry reports, and death certificates.

### *TP53* polymorphisms

DNA was extracted from white blood cells by using the standard procedures of phenol and chloroform extraction. *TP53* rs1042522 and rs12947788 were genotyped using the Sequenom iPLEX MassArray (Sequenom, Inc., San Diego, CA). We performed iPLEX genotyping through MALDI-TOF spectroscopy by using the Sequenom MassARRAY platform and iPLEX GOLD chemistry. We used 10 ng of genomic DNA as a template and added a PCR mix containing Qiagen HotStarTaq to the template. Shrimp alkaline phosphatase and primer extension steps were performed using the Sequenom protocol and reagents. Primers were obtained from Integrated DNA Technologies (OH, USA). Assays were designed using the MassARRAY Assay Design, Version 3.1 (Sequenom). Raw genotype data were visualized and processed using the MassARRAY Typer software, Version 4.0. Genotyping was repeated on 10% of the samples for quality control.

### Statistical analysis

Descriptive statistics was used to describe the sociodemographic characteristics and lifestyle factors of the MMR gene germline mutation carriers. The risk was considered to begin at birth and end at the diagnosis of CRC, death, or loss to follow-up, whichever occurred first. The MMR gene germline mutation carriers who did not receive a diagnosis of CRC were censored at the date of their last known contact or in February 2012.

The genotype distribution of each *TP53* polymorphism was examined using the Hardy–Weinberg equilibrium, and differences between expected and observed frequencies were tested for statistical significance by using Pearson’s chi-square test. The identification of the MMR gene germline mutation carriers was not randomly ascertained. Thus, we adjusted for this nonrandom ascertainment by using a weighted cohort analysis approach, as described by Antoniou et al. [22]. A weighted Cox proportional hazard model was used to calculate hazard ratios (HRs) and 95% confidence intervals (CIs) to determine the association of *TP53* polymorphisms with CRC development. Mutation in MMR genes (*MLH1* or *MSH2*), sex, colonoscopy screening, and year of birth (<1940, 1940–1949, 1950–1959, 1960–1969, 1970–1979, and >1980) were adjusted as potential confounding factors in the multivariable Cox model. A robust sandwich covariance estimation model was used to adjust for within-cluster and within-family correlations [23].

Multiplicative interactions between *TP53* polymorphisms and lifestyle factors such as cigarette smoking and alcohol, tea, and coffee consumption were assessed using HRs for interactions by including their product terms in the Cox proportional model. We used the PHASE software [24] to reconstruct haplotypes from the genotypes of rs1042522 and rs12947788 polymorphisms for each MMR gene germline mutation carrier. The PHASE software is a Bayesian statistical method used to reconstruct haplotypes from population genotype data. The survival curves for each *TP53* polymorphism were plotted using the Kaplan–Meier method. A P value of <0.05 was considered statistically significant, and all analyses were performed using the Statistical Analysis Software (SAS) package (Version 9.4 for Windows; SAS Institute, Inc., Cary, NC, USA). All statistical tests were two sided.

## Results

Germline mutations in *MLH1* and *MSH2* were identified from the Amsterdam II criteria families, and the diagnosis of pathologically confirmed CRC was the outcome of interest. The study population comprised 184 *MLH1* and 76 *MSH2* germline mutation carriers from 62 families, accounting for a total person time of 10,803 years. During the follow-up period, 120 (46.2%) carriers developed histologically confirmed CRC. Of these carriers, 74 (61.7%) were family members of the probands, 65 (54.2%) were women, and 96 (80.0%) were *MLH1* germline mutation carriers (Table 1). The median age at CRC diagnosis was 44 years. Of the carriers, 186 (71.5%) never smoked cigarettes and 181 (69.6%), 102 (39.2%), and 175 (67.6%) never consumed alcohol, tea, and coffee, respectively. Furthermore, approximately 36% of the patients with CRC were born between 1950 and 1959. Approximately 135 (96.4%) of the family members were CRC free at the last date of follow-up, and colonoscopy was performed to confirm the CRC-free status.

**Table 1:** Characteristics of MMR gene germline mutation carriers.

Variables	Total cohort (n = 260) n (%)	CRC cases (n = 120) n (%)
Age at diagnosis, median (IQR)^a^	44 (37–52)	
Sex		
Female	139 (53.5)	65 (54.2)
Male	121 (46.5)	55 (45.8)
Role in study		
Proband	51 (19.6)	46 (38.3)
Family member	209 (80.4)	74 (61.7)
MMR gene mutated		
*MLH1*	184 (70.7)	96 (80.0)
*MSH2*	76 (29.3)	24 (20.0)
Cigarette smoking		
Never	186 (71.5)	86 (71.7)
Ever	74 (28.5)	34 (28.3)
Alcohol drinking		
Never	181 (69.6)	91 (75.8)
Ever	79 (30.4)	29 (24.2)
Tea consumption		
Never	102 (39.2)	62 (51.7)
Ever	158 (60.8)	58 (48.3)
Coffee consumption		
Never	175 (67.3)	95 (79.2)
Ever	85 (32.7)	25 (20.8)
Birth cohort		
<1940	25 (9.60)	22 (18.3)
1940–1949	26 (10.0)	17 (14.2)
1950–1959	65 (25.0)	43 (35.8)
1960–1969	50 (19.3)	26 (21.7)
1970–1979	56 (21.5)	12 (10.0)
>1980	38 (14.6)	-

IQR: interquartile range

^a^IQR (25^th^–75^th^ percentiles)

**Fig 1:**
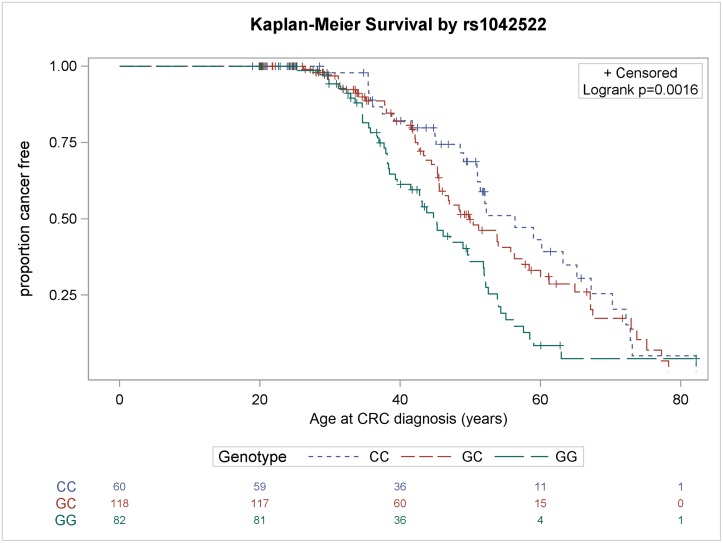
Kaplan–Meier CRC-free survival according to the *TP53* rs1042522 single-nucleotide polymorphism (SNP). The figure demonstrates an early onset of CRC in MMR gene germline mutation carriers with homozygous wild type GG genotype as compared with the heterozygous GC and homozygous variant CC genotype.

**Fig 2:**
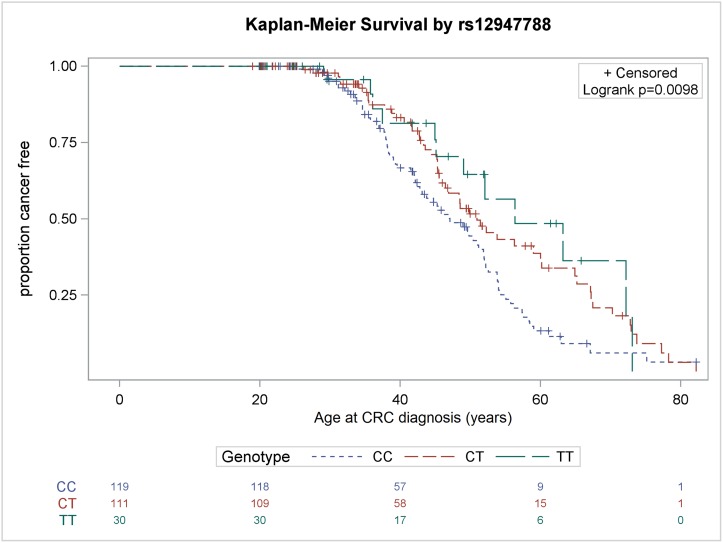
Kaplan–Meier CRC-free survival according to the *TP53* rs12947788 SNP. The figure demonstrates an early onset of CRC in MMR germline mutation carriers with homozygous wild type CC genotype as compared with the heterozygous CT and homozygous variant TT genotype.

As presented in Fig 1, the results of the Kaplan–Meier CRC-free survival analysis and log-rank test revealed that the GG genotype of the rs1042522 polymorphism was significantly associated with an earlier onset of CRC compared with other genotypes (P = 0.0016). (Fig 1. Kaplan–Meier CRC-free survival according to the *TP53* rs1042522 single-nucleotide polymorphism [SNP]. The figure demonstrates an early onset of CRC in MMR gene germline mutation carriers with homozygous wild type GG genotype as compared with the heterozygous GC and homozygous variant CC genotype). The median age at CRC diagnosis for the GG, GC, and CC genotypes was 39, 45, and 51 years, respectively. In addition, the results of the log-rank test revealed that compared with other genotypes, the CC genotype of the rs12947788 polymorphism was associated with a significantly earlier onset of CRC (P = 0.0098, Fig 2). (Fig 2. Kaplan–Meier CRC-free survival according to the *TP53* rs12947788 SNP. The figure demonstrates an early onset of CRC in MMR germline mutation carriers with homozygous wild type CC genotype as compared with the heterozygous CT and homozygous variant TT genotype). The median age at CRC diagnosis for the CC, CT, and TT genotypes was 42, 45, and 47 years, respectively.

Table 2 shows the association of *TP53* polymorphisms with CRC development in the patients with Lynch syndrome. Both *TP53* polymorphisms rs1042522 and rs12947788 followed the Hardy–Weinberg Equilibrium (P = 0.17 and 0.60, respectively). The adjusted HR revealed that the heterozygote variant GC and homozygote variant CC were associated with a decreased CRC risk for the rs1042522 polymorphism (HR = 0.35, 95% CI = 0.14–0.86 and HR = 0.28, 95% CI = 0.13–0.57, respectively). Further analysis revealed an association of the dominant model with a decreased CRC risk (HR = 0.32, 95% CI = 0.15–0.67). For the rs12947788 polymorphism, the heterozygote variant CT and homozygote variant TT were associated with a decreased CRC risk (HR = 0.20, 95% CI = 0.08–0.46 and HR = 0.25, 95% CI = 0.09–0.65, respectively). The dominant model of the rs12947788 polymorphism was significantly associated with a decreased CRC risk (HR = 0.21, 95% CI = 0.09–0.46).

**Table 2:** Hazard ratios of *TP53* polymorphisms associated with CRC risk among MMR germline mutation carriers.

Genotype	Total cohort	Person -years	CRC cases	Crude		Adjusted	
				HR (95% CI)	P value	HR (95% CI)^a^	P value
***TP53* rs1042522**							
GG	82	3273	44	1.00		1.00	
GC	118	4883	52	0.53 (0.21–1.32)	0.175	**0.35 (0.14–0.86)**	**0.022**
CC	60	2647	24	0.46 (0.20–1.03)	0.061	**0.28 (0.13–0.57)**	**0.001**
Dominant model							
GG	82	3273	44	1.00		1.00	
GC+CC	178	7530	76	0.50 (0.22–1.11)	0.091	**0.32 (0.15–0.67)**	**0.002**
Recessive model							
GG+GC	200	8155	96	1.00		1.00	
CC	60	2647	24	0.72 (0.37–1.38)	0.319	0.60 (0.33–1.08)	0.092
***TP53* rs12947788**							
CC	119	4874	64	1.00		1.00	
CT	111	4629	46	**0.35 (0.17–0.71)**	**0.004**	**0.20 (0.08–0.46)**	**0.001**
TT	30	1300	10	**0.46 (0.22–0.96)**	**0.038**	**0.25 (0.09–0.65)**	**0.004**
Dominant model							
CC	119	4874	64	1.00		1.00	
CT+TT	141	5929	56	**0.37 (0.19–0.72)**	**0.003**	**0.21 (0.09–0.46)**	**0.001**
Recessive model							
CC+CT	230	9503	110	1.00		1.00	
TT	30	1300	10	0.85 (0.47–1.52)	0.589	0.82 (0.46–1.45)	0.491

^a^ Adjusted for sex, colonoscopy screening, date of birth, familial clustering, and specific mutated MMR gene

We performed haplotype analysis to evaluate the effect of the combination of these SNPs on CRC risk in the patients with Lynch syndrome. As depicted in Table 3, compared with the most common haplotype GC, the haplotype CT was significantly associated with a decreased CRC risk (HR = 0.26, 95% CI = 0.11–0.59). However, the haplotypes GT (HR = 0.54, 95% CI = 0.12–2.34) and CC (HR = 1.05, 95% CI = 0.58–1.89) were not significantly associated with a decreased CRC risk. Furthermore, no significant multiplicative interaction was observed between *TP53* polymorphisms and lifestyle factors (S1 Table).

**Table 3:** Haplotype analysis for *TP53* polymorphism and CRC risk among MMR germline mutation carriers.

Haplotypes	Allele frequency n (%)	Crude		Adjusted	
		HR (95% CI)	P value	HR (95% CI)^a^	P value
**rs1042522/rs12947788**					
G/C	278 (53.5)	1.00		1.00	
G/T	4 (0.8)	0.47 (0.10–2.14)	0.331	0.54 (0.12–2.34)	0.413
C/C	72 (13.8)	1.17 (0.65–2.10)	0.611	1.05 (0.58–1.89)	0.879
C/T	166 (31.9)	**0.41 (0.21–0.82)**	**0.011**	**0.26 (0.11–0.59)**	**0.001**

^a^ Adjusted for sex, colonoscopy screening, date of birth, familial clustering, and specific mutated MMR gene

## Discussion

The results of the present study revealed that the GC and CC genotypes of rs1042522 and the CT and TT genotypes of rs12947788 were associated with a decreased CRC risk in the patients with Lynch syndrome in Taiwan. *TP53* rs1042522 and rs12947788 are located in exon 4 and intron 7 of chromosome 17, respectively [25], and have a critical role of modulating defense against cancer development. Studies have investigated the association of *TP53* polymorphisms with CRC risk, and the results reported thus far have been inconsistent [17, 19, 26, 27, 28]. This inconsistency may be due to differences among study populations with different allele frequencies of *TP53* polymorphisms. In the Chinese population, the C and T alleles are minor alleles with allelic frequencies of 0.431 and 0.327 for rs1042522 and rs12947788, respectively [29, 30], which are different from those in Caucasians. Moreover, these two polymorphisms are in high linkage disequilibrium (D’ = 0.95).

Studies have reported an association of people with a Pro allele in their *TP53* Arg72Pro (rs1042522) polymorphism with a decreased risk of sporadic CRC [31, 32, 33]. However, a case–control study reported no association of the *TP53* polymorphism with CRC in patients with Lynch syndrome [34]. The protective effect of the C allele observed in our study can be attributed to its high efficiency in maintaining genomic integrity by arresting cells having mutated or damaged DNA in the G1 phase of the cell cycle to enable the repair mechanism or induce the apoptosis pathway [35]. The balance between cell cycle arrest and induced apoptosis depends on the severity of DNA damage [7]. When DNA is severely damaged and cannot be repaired, cells may directly induce the apoptosis pathway. However, less damaged cells may undergo the repair mechanism after being arrested in the G1 phase of the cell cycle. Thus, C allele carriers might have a low CRC risk. In addition, Tan et al. suggested that the variant Pro allele of *TP53* Arg72Pro is associated with a decreased risk of CRC because of its high efficiency in cell cycle arrest [32].

To the best of our knowledge, this is the first study reporting an association of the rs12947788 polymorphism with CRC in patients with Lynch syndrome. In this study, we observed that compared with MMR gene germline mutation carriers with the homozygote CC genotype, those with CT and TT genotypes were associated with a decreased CRC risk. However, the biological mechanism underlying the protective effect of the minor T allele of rs12947788 against CRC in patients with Lynch syndrome has rarely been investigated and thus remains unclear. A case–control study conducted in the Czech Republic reported that the minor T allele and heterozygote and homozygote variant genotypes of rs12947788 were associated with a decreased CRC risk [36]; this finding is consistent with that of our study. However, the results of Polakova et al. were not significant probably because of the low frequency of the minor allele T in both case and control groups.

Our study results revealed that carriers with the homozygote GG genotype of rs1042522 developed CRC 12 years earlier in life than those with the variant CC genotype did. This finding confirmed that the G allele is less effective in preventing CRC development. However, Jones et al. reported that Lynch syndrome carriers with a heterozygous genotype of rs1042522 developed CRC 13 years earlier in life than those with the G allele did [19]. This discrepancy can be attributed to the allele frequency variation of rs1042522 between Caucasian and Chinese populations. Furthermore, we observed that compared with the TT genotype, the CC genotype of rs12947788 was associated with a 4-year earlier onset of CRC.

Compared with single-SNP analysis, haplotype-based analysis is more powerful and has higher statistical power [36]. In this study, we determined that the haplotype C/T was protective as compared to the common haplotype G/C. However, no significant association of haplotypes containing C/T with cancer risk was observed [36, 37, 38]. This discrepancy can be attributed to the low frequencies of the C/T haplotype observed in these studies [36, 37, 38]. The identification of protective haplotypes as a host genetic factor can facilitate understanding the pathogenesis of CRC. However, additional studies are required to confirm this association.

A multiplicative interaction between *TP53* polymorphisms and lifestyle factors in patients with Lynch syndrome has not yet been reported. In this study, no significant multiplicative interactions were observed between *TP53* polymorphisms and lifestyle factors. This finding is consistent with that of a previous study that reported a nonsignificant multiplicative and additive interaction between *TP53* polymorphisms and lifestyle factors in patients with lung cancer [39]. Some case–control studies in India have reported a significant interaction between *TP53* polymorphisms and tobacco smoking in patients with oral and stomach cancers [40, 41]. However, these studies did not indicate P values for interaction measures and they did not include patients with Lynch syndrome; thus, drawing a conclusion from these studies is difficult.

The major strengths of our study are that all study participants were confirmed to have hereditary MMR gene germline mutations, and all CRC diagnoses were confirmed histologically. However, this study was limited by our inability to test other MMR gene germline mutations associated with Lynch syndrome, including *MSH6*, *PMS2*, and *EPCAM* germline mutations. The diagnosis of CRC was confounded by the time to access medical care services. Moreover, approximately 54% of the mutation carriers were not willing to be followed up for their recent morbidity status at least once since recruitment; hence, we likely failed to record some newly developed cancer cases. In addition, we excluded 41 patients with Lynch syndrome because their *TP53* genetic polymorphism results were unavailable. Nevertheless, demographic characteristics did not differ between included and excluded patients. In addition, we could not examine other *TP53* polymorphisms such as polymorphisms in intron 2 (rs1642785) and intron 3 (rs17878362).

In conclusion, the study results revealed that the variant C allele of rs1042522 and the variant T allele of rs12947788 were associated with a decreased CRC risk in patients with MMR gene germline mutations in Taiwan. In addition, the results revealed that MMR gene germline mutation carriers harboring rs1042522 GG and rs12947788 CC genotypes have an increased risk of early-onset CRC. Therefore, we suggest that genetic screening and regular colonoscopy be started early, particularly in patients with Lynch syndrome harboring risk alleles.

## Supporting Information

S1 Table Interaction between *TP53* polymorphisms and lifestyle factors and CRC risk among MMR germline mutation carriers. (DOCX)

## References

[1] De Jong AE, Morreau H, Van Puijenbroek M, Eilers PH c, Wijnen J, Nagengast FM, et al The role of mismatch repair gene defects in the development of adenomas in patients with HNPCC. Gastroenterology. 2004; 126: 42–48. 14699485 10.1053/j.gastro.2003.10.043

[2] Evaluation of Genomic Applications in Practice and Prevention (EGAPP) Working Group. Recommendations from the EGAPP Working Group: genetic testing strategies in newly diagnosed individuals with colorectal cancer aimed at reducing morbidity and mortality from Lynch syndrome in relatives. Genet Med. 2009; 11: 35–41. 10.1097/GIM.0b013e31818fa2ff 19125126 PMC2743612

[3] de la Chapelle A. Genetic predisposition to colorectal cancer. Nat Rev Cancer. 2004; 4: 769–780. 10.1038/nrc1453 15510158

[4] Watson P, Vasen HFA, Mecklin J-P, Bernstein I, Aarnio M, Järvinen HJ, et al The risk of extra-colonic, extra-endometrial cancer in the Lynch syndrome. Int J Cancer. 2008; 123: 444–449. 10.1002/ijc.23508 18398828 PMC2627772

[5] Fu L, Sheng J, Li X, Jin P, Mu H, Han M, et al Mismatch repair gene mutation analysis and colonoscopy surveillance in Chinese Lynch syndrome families. Cell Oncol Dordr. 2013; 36: 225–231. 10.1007/s13402-013-0130-z 23640085 PMC13012685

[6] Xu H, el-Gewely MR. P53-responsive genes and the potential for cancer diagnostics and therapeutics development. Biotechnol Annu Rev. 2001; 7: 131–164. 11686042 10.1016/s1387-2656(01)07035-1

[7] Meek DW. The p53 response to DNA damage. DNA Repair. 2004; 3: 1049–1056. 10.1016/j.dnarep.2004.03.027 15279792

[8] Whibley C, Pharoah PDP, Hollstein M. p53 polymorphisms: cancer implications. Nat Rev Cancer. 2009; 9: 95–107. 10.1038/nrc2584 19165225

[9] Campbell PJ, Yachida S, Mudie LJ, Stephens PJ, Pleasance ED, Stebbings LA, et al The patterns and dynamics of genomic instability in metastatic pancreatic cancer. Nature. 2010; 467: 1109–1113. 10.1038/nature09460 20981101 PMC3137369

[10] Bartkowiak K, Riethdorf S, Pantel K. The Interrelating Dynamics of Hypoxic Tumor Microenvironments and Cancer Cell Phenotypes in Cancer Metastasis. Cancer Microenviron. 2012; 5: 59–72. 10.1007/s12307-011-0067-6 21626313 PMC3343196

[11] Själander A, Birgander R, Athlin L, Stenling R, Rutegård J, Beckman L, et al P53 germ line haplotypes associated with increased risk for colorectal cancer. Carcinogenesis. 1995; 16: 1461–1464. 7614678 10.1093/carcin/16.7.1461

[12] Sayhan N, Yazici H, Budak M, Bitisik O, Dalay N. P53 codon 72 genotypes in colon cancer. Association with human papillomavirus infection. Res Commun Mol Pathol Pharmacol. 2001; 109: 25–34. 11458982

[13] Gemignani F, Moreno V, Landi S, Moullan N, Chabrier A, Gutiérrez-Enríquez S, et al A TP53 polymorphism is associated with increased risk of colorectal cancer and with reduced levels of TP53 mRNA. Oncogene. 2003; 23: 1954–1956. 10.1038/sj.onc.1207305 14647431

[14] Pérez LO, Abba MC, Dulout FN, Golijow CD. Evaluation of p53 codon 72 polymorphism in adenocarcinomas of the colon and rectum in La Plata, Argentina. World J Gastroenterol WJG. 2006; 12: 1426–1429. 10.3748/wjg.v12.i9.1426 16552814 PMC4124323

[15] Zhu ZZ, Wang AZ, Jia HR, Jin XX, He XL, Hou LF, et al Association of the TP53 codon 72 polymorphism with colorectal cancer in a Chinese population. Jpn J Clin Oncol. 2007; 37: 385–390. 10.1093/jjco/hym034 17599946

[16] Song HR, Kweon SS, Kim HN, Piao JM, Yun WJ, Choi JS, et al p53 codon 72 polymorphism in patients with gastric and colorectal cancer in a Korean population. Gastric Cancer. 2011; 14: 242–247. 10.1007/s10120-011-0034-4 21461655

[17] Talseth BA, Meldrum C, Suchy J, Kurzawski G, Lubinski J, Scott RJ. Age of diagnosis of colorectal cancer in HNPCC patients is more complex than that predicted by R72P polymorphism in TP53. Int J Cancer. 2006; 118: 2479–2484. 10.1002/ijc.21661 16353134

[18] Sotamaa K, Liyanarachchi S, Mecklin JP, Järvinen H, Aaltonen LA, Peltomäki P, et al p53 Codon 72 and MDM2 SNP309 Polymorphisms and Age of Colorectal Cancer Onset in Lynch Syndrome. Clin Cancer Res. 2005; 11: 6840–6844. 10.1158/1078-0432.CCR-05-1139 16203772

[19] Jones JS, Chi X, Gu X, Lynch PM, Amos CI, Frazier ML. p53 Polymorphism and Age of Onset of Hereditary Nonpolyposis Colorectal Cancer in a Caucasian Population. Clin Cancer Res. 2004; 10: 5845–5849. 10.1158/1078-0432.CCR-03-0590 15355915

[20] Tang R, Hsiung C, Wang JY, Lai CH, Chien HT, Chiu LL, et al Germ line MLH1 and MSH2 mutations in Taiwanese Lynch syndrome families: characterization of a founder genomic mutation in the MLH1 gene. Clin Genet. 2009; 75: 334–345. 10.1111/j.1399-0004.2009.01162.x 19419416

[21] Kamiza AB, Hsieh LL, Tang R, Chien HT, Lai CH, Chiu LL, et al Risk Factors Associated with Colorectal Cancer in a Subset of Patients with Mutations in MLH1 and MSH2 in Taiwan Fulfilling the Amsterdam II Criteria for Lynch Syndrome. PloS One. 2015; 10: e0130018 10.1371/journal.pone.0130018 26053027 PMC4460082

[22] Antoniou AC, Goldgar DE, Andrieu N, Chang-Claude J, Brohet R, Rookus MA, et al A weighted cohort approach for analysing factors modifying disease risks in carriers of high-risk susceptibility genes. Genet Epidemiol. 2005; 29: 1–11. 10.1002/gepi.20074 15880399

[23] Williams RL. A note on robust variance estimation for cluster-correlated data. Biometrics. 2000; 56: 645–646. 10877330 10.1111/j.0006-341x.2000.00645.x

[24] Stephens M, Smith NJ, Donnelly P. A new statistical method for haplotype reconstruction from population data. Am J Hum Genet. 2001; 68: 978–989. 10.1086/319501 11254454 PMC1275651

[25] Naccarati A, Polakova V, Pardini B, Vodickova L, Hemminki K, Kumar R, et al Mutations and polymorphisms in TP53 gene—an overview on the role in colorectal cancer. Mutagenesis. 2012; 27: 211–218. 10.1093/mutage/ger067 22294769

[26] Chen J, Etzel CJ, Amos CI, Zhang Q, Viscofsky N, Lindor NM, et al Genetic variants in the cell cycle control pathways contribute to early onset colorectal cancer in Lynch syndrome. Cancer Causes Control. 2009; 20: 1769–1777. 10.1007/s10552-009-9416-x 19690970 PMC3917505

[27] Dahabreh IJ, Linardou H, Bouzika P, Varvarigou V, Murray S. TP53 Arg72Pro polymorphism and colorectal cancer risk: a systematic review and meta-analysis. Cancer Epidemiol Biomark Prev Publ Am Assoc Cancer Res Cosponsored Am Soc Prev Oncol. 2010; 19: 1840–1847. 10.1158/1055-9965.EPI-10-0156 20615891

[28] Tang NP, Wu YM, Wang B, Ma J. Systematic review and meta-analysis of the association between P53 codon 72 polymorphism and colorectal cancer. Eur J Surg Oncol l. 2010; 36: 431–438. 10.1016/j.ejso.2010.03.010 20363586

[29] Sucheston L, Witonsky DB, Hastings D, Yildiz O, Clark VJ, Di Rienzo A, et al Natural selection and functional genetic variation in the p53 pathway. Hum Mol Genet. 2011; 20: 1502–1508. 10.1093/hmg/ddr028 21266458 PMC3063984

[30] Berggren P, Kumar R, Steineck G, Ichiba M, Hemminki K. Ethnic variation in genotype frequencies of a p53 intron 7 polymorphism. Mutagenesis. 2001; 16: 475–478. 11682637 10.1093/mutage/16.6.475

[31] Zahary MN, Ahmad Aizat AA, Kaur G, Yeong Yeh L, Mazuwin M, Ankathil R. Polymorphisms of cell cycle regulator genes CCND1 G870A and TP53 C215G: Association with colorectal cancer susceptibility risk in a Malaysian population. Oncol Lett. 2015; 10: 3216–3222. 10.3892/ol.2015.3728 26722315 PMC4665742

[32] Tan XL, Nieters A, Hoffmeister M, Beckmann L, Brenner H, Chang-Claude J. Genetic polymorphisms in TP53, nonsteroidal anti-inflammatory drugs and the risk of colorectal cancer: evidence for gene-environment interaction? Pharmacogenet Genomics. 2007; 17: 639–645. 10.1097/FPC.0b013e3280d5121c 17622940

[33] Dakouras A, Nikiteas N, Papadakis E, Perakis M, Valis D, Rallis G, et al p53Arg72 Homozygosity and its Increased Incidence in Left-sided Sporadic Colorectal Adenocarcinomas, in a Greek-Caucasian Population. Anticancer Res. 2008; 28: 1039–1043. 18507052

[34] Krüger S, Bier A, Engel C, Mangold E, Pagenstecher C, von Knebel Doeberitz M, et al The p53 codon 72 variation is associated with the age of onset of hereditary non-polyposis colorectal cancer (HNPCC). J Med Genet. 2005; 42: 769–773. 10.1136/jmg.2004.028506 16199549 PMC1735929

[35] Pim D, Banks L. p53 polymorphic variants at codon 72 exert different effects on cell cycle progression. Int J Cancer. 2004; 108: 196–199. 10.1002/ijc.11548 14639602

[36] Polakova V, Pardini B, Naccarati A, Landi S, Slyskova J, Novotny J, et al Genotype and haplotype analysis of cell cycle genes in sporadic colorectal cancer in the Czech Republic. Hum Mutat. 2009; 30: 661–668. 10.1002/humu.20931 19224585

[37] Sprague BL, Trentham-Dietz A, Garcia-Closas M, Newcomb PA, Titus-Ernstoff L, Hampton JM, et al Genetic variation in TP53 and risk of breast cancer in a population-based case control study. Carcinogenesis. 2007; 28: 1680–1686. 10.1093/carcin/bgm097 17449902

[38] Naccarati A, Pardini B, Polakova V, Smerhovsky Z, Vodickova L, Soucek P, et al Genotype and haplotype analysis of TP53 gene and the risk of pancreatic cancer: an association study in the Czech Republic. Carcinogenesis. 2010; 31: 666–670. 10.1093/carcin/bgq032 20110284

[39] Li Y, Chang SC, Niu R, Liu L, Crabtree-Ide CR, Zhao B, et al TP53 genetic polymorphisms, interactions with lifestyle factors and lung cancer risk: a case control study in a Chinese population. BMC Cancer. 2013; 13: 607 10.1186/1471-2407-13-607 24369748 PMC3877976

[40] Ihsan R, Devi TR, Yadav DS, Mishra AK, Sharma J, Zomawia E, et al Investigation on the role of p53 codon 72 polymorphism and interactions with tobacco, betel quid, and alcohol in susceptibility to cancers in a high-risk population from North East India. DNA Cell Biol. 2011; 30: 163–171. 10.1089/dna.2010.1119 21043833

[41] Malakar M, Devi KR, Phukan RK, Kaur T, Deka M, Puia L, et al p53 codon 72 polymorphism interactions with dietary and tobacco related habits and risk of stomach cancer in Mizoram, India. Asian Pac J Cancer Prev APJCP. 2014; 15: 717–723. 24568485 10.7314/apjcp.2014.15.2.717

