# Supplementary material for: TP53 Polymorphisms and Colorectal Cancer Risk in Patients with Lynch Syndrome in Taiwan: A Retrospective Cohort Study
Source: PLoS One. 2016 Dec 1;11(12):e0167354. doi: 10.1371/journal.pone.0167354 (PMC5131981; doi:10.1371/journal.pone.0167354)
Supplement: S1 Table — (DOCX) [file pone.0167354.s001.docx]

| S1 Table. Interaction between *TP53* polymorphism and lifestyle factors and risk of CRC among MMR germline mutation carriers. | | | | | |
| --- | --- | --- | --- | --- | --- |
| Lifestyle factors | Genotype | No | CRC cases | HR (95% CI)^a^ | P value |
| **Cigarette smoking** | **rs1042522** |  |  |  |  |
| Never | GG | 61 | 35 | 1.00 |  |
| Never | GC+CC | 125 | 51 | 0.62 (0.29–1.31) | 0.208 |
| Ever | GG | 21 | 9 | 1.00 |  |
| Ever | GC+CC | 53 | 25 | **0.56 (0.32–0.98)** | **0.043** |
| Interaction |  |  |  |  | 0.331 |
| **Alcohol drinking** | **rs1042522** |  |  |  |  |
| Ever | GG | 24 | 11 | 1.00 |  |
| Ever | GC+CC | 55 | 18 | 0.46 (0.20–1.05) | 0.066 |
| Never | GG | 58 | 33 | 1.00 |  |
| Never | GC+CC | 123 | 58 | 0.64 (0.31–1.32) | 0.231 |
| Interaction |  |  |  |  | 0.402 |
| **Tea consumption** | **rs1042522** |  |  |  |  |
| Ever | GG | 51 | 25 | 1.00 |  |
| Ever | GC+CC | 107 | 33 | **0.53 (0.29–0.95)** | **0.032** |
| Never | GG | 31 | 19 | 1.00 |  |
| Never | GC+CC | 71 | 43 | 0.94 (0.50–1.75) | 0.847 |
| Interaction |  |  |  |  | 0.974 |
| **Coffee consumption** | **rs1042522** |  |  |  |  |
| Ever | GG | 25 | 10 | 1.00 |  |
| Ever | GC+CC | 60 | 15 | 0.52 (0.26–1.02) | 0.058 |
| Never | GG | 57 | 34 | 1.00 |  |
| Never | GC+CC | 118 | 61 | 0.58 (0.28–1.18) | 0.136 |
| Interaction |  |  |  |  | 0.776 |
| **Cigarette smoking** | **rs12947788** |  |  |  |  |
| Never | CC | 89 | 50 | 1.00 |  |
| Never | CT+TT | 97 | 36 | **0.37 (0.19–0.69)** | **0.002** |
| Ever | CC | 30 | 14 | 1.00 |  |
| Ever | CT+TT | 44 | 20 | **0.54 (0.31–0.95)** | **0.032** |
| Interaction |  |  |  |  | 0.259 |
| **Alcohol drinking** | **rs12947788** |  |  |  |  |
| Ever | CC | 35 | 13 | 1.00 |  |
| Ever | CT+TT | 44 | 16 | **0.36 (0.16–0.78)** | **0.011** |
| Never | CC | 84 | 51 | 1.00 |  |
| Never | CT+TT | 97 | 40 | **0.36 (0.16–0.79)** | **0.011** |
| Interaction |  |  |  |  | 0.611 |
| **Tea consumption** | **rs12947788** |  |  |  |  |
| Ever | CC | 73 | 32 | 1.00 |  |
| Ever | CT+TT | 85 | 26 | **0.47 (0.25–0.86)** | **0.016** |
| Never | CC | 46 | 32 | 1.00 |  |
| Never | CT+TT | 56 | 30 | 0.63 (0.34–1.16) | 0.143 |
| Interaction |  |  |  |  | 0.847 |
| **Coffee consumption** | **rs12947788** |  |  |  |  |
| Ever | CC | 33 | 11 | 1.00 |  |
| Ever | CT+TT | 52 | 14 | **0.44 (0.21–0.92)** | **0.028** |
| Never | CC | 86 | 53 | 1.00 |  |
| Never | CT+TT | 89 | 42 | **0.38 (0.18–0.77)** | **0.008** |
| Interaction |  |  |  |  | 0.546 |
| ^a^ Adjusted for sex, colonoscopy screening, date of birth, familial clustering, and specific mutated MMR gene | | | | | |
